# Utilization of Existing Human Kinase Inhibitors as Scaffolds in the Development of New Antimicrobials

**DOI:** 10.3390/antibiotics12091418

**Published:** 2023-09-07

**Authors:** Monika I. Konaklieva, Balbina J. Plotkin

**Affiliations:** 1 Department of Chemistry, American University, Washington, DC 20016, USA; 2 Department of Microbiology and Immunology, Midwestern University, Downers Grove, IL 60515, USA; bplotk@midwestern.edu

**Keywords:** antimicrobials, human kinase inhibitors, drug resistance, drug repurposing

## Abstract

The prevalence and continuing expansion of drug resistance, both in clinical and community settings represents a major challenge for current antimicrobial therapy. The different approaches for addressing this challenge include (1) identification of novel antibacterials by repurposing of existing drugs originally that historically target host proteins; and (2) effect target switching through modification of existing antimicrobials. The focus of this manuscript is on these drug discovery strategies, with utility for development of new antimicrobials with different modes of action.

## 1. Introduction

In the 2015 Global Action Plan, the World Health Organization (WHO) emphasizes the urgent need to address antimicrobial resistance [1]. Coinciding with this rise in threat to health caused by multi-drug resistant (MDR) organisms, is the need for new antibiotics as stressed by the WHO analysis of the antibacterial drug pipeline which reports an alarming lack of new drugs in development [1]. The need for new drugs is further illustrated by the 2017 Centers for Disease Control and Prevention (CDC) report of 220 cases of virtually untreatable bacteria [2]. Ironically, these alarming reports come approximately 100 years since the idea of anti-infective drug development was first proposed. Unfortunately, development of effective antimicrobial drugs is a profound scientific challenge since they have different physicochemical properties, as compared to the majority of other drug classes. Moreover, there is the requirement for an expeditious reporting system that bridges preparation steps of a given synthetic organic entity with cell morphology/gene expression changes induced by the drug if there is to be future success in the development of successful antimicrobials. The aforementioned challenges require re-thinking of the strategies for antimicrobial drug design. One such strategy is drug repurposing, i.e., evaluation of drugs as antimicrobials from the pool of existing drugs/drug candidates originally designed and tested for uses other than as antimicrobials. This strategy has several advantages, amongst which is lowering the cost of developing a novel antimicrobial that could lose effectiveness in the short term due to MDR development [3]. This review will concentrate on the most recent decade or so of repurposing of eukaryotic (human) kinase inhibitors (HumKIs) as antimicrobials. The HumKIs discussed hereinafter are either FDA-approved, or are in the public domain, and have demonstrated activity as inhibitors of bacterial enzymes (mainly but not limited to bacterial kinases). For more in-depth reviews on bacterial kinases and their inhibitors, the reader is directed elsewhere [4,5,6].

Historically, thirty years ago, kinases were considered “undruggable” targets, while today kinases are the target of at least a quarter of the pharmaceutical industries’ drug innovation efforts [7,8,9]. In fact, they are considered one of the main targets for numerous chronic progressive disorders, including cancer. Mechanistically, kinases transfer phosphate groups from ATP to specific proteins or small biomolecules, such as lipids and carbohydrates. Thus, kinases phosphorylate molecules that regulate diverse cellular processes, including cellular growth, replication, differentiation, motility, and programmed cell death [7]. Since phosphorylation imposes structural changes on the substrate it affects, due to its capacity to associate with other molecules, its subcellular localization, and/or its catalytic activity, kinase inhibition has motivated large investments in drug discovery programs [8]. This has allowed for the public domain availability of more than five thousand protein kinase structures, in addition to the proprietary structures within the pharmaceutical industry. The accessibility of this information promotes further structure-function based drug development which has led to approximately 180 orally effective protein kinase inhibitors that are in clinical testing world-wide, with an additional 72 currently FDA approved [9]. These FDA-approved drugs include some that target protein-serine/threonine protein kinases, some that block nonreceptor protein-tyrosine kinases, and others that are dual target protein kinases (MEK1/2), with the majority targeting receptor protein/tyrosine kinases. From the total 72 FDA approved drugs, 62 are developed/prescribed for treatment of neoplasms [9]. Oral administration of the FDA-approved drugs is effective for all the kinase-based drugs with the exceptions of netarsudil, trilaciclib, and temsirolimus [9].

It is interesting to note that more than 24 of the FDA approved HumKIs are multikinase inhibitors. It is not inconceivable to assume that there will be more multikinase agonists amongst the rest of the 72 HumKIs since the specificity of most of the HumKIs has not been determined [9]. While the pros and cons of drug specificity vs. polypharmacology are difficult to predict/evaluate for each drug [9], the off-target effect is an enzyme phenomenon. In recent years, several types of deviations from the widely accepted view of an enzyme’s (strict) specificity to its ligand have been observed and are referred to as “promiscuous behavior” [10]. This enzyme behavior is demonstrated by (i) allowing for several biochemical functions to be performed by the same enzyme, (ii) to be the origin of new proteins via gene duplication, and (iii) lack a preformed binding site due to the intrinsic disorder for both prokaryotic and eukaryotic proteins [10]. It is reported that in many protein families significant structural homology exists, which allows for ligands to display promiscuity [10]. This promiscuity provides pathogens with opportunities to evade antimicrobial drug activity via ligand binding to enzymes from different kingdoms (Eukaryota and Prokaryota), with cross inhibition of human and microbial kinases as an example. Exploring the ability of a ligand (drug) to bind to an unrelated target (receptor) falls under the rubric of drug repurposing. One of the first decades old examples of repurposing is the use of aspirin as a blood thinner. Fortunately, the current availability of information about pharmacokinetics, toxicity, and possible side effects of a drug facilitates ligand repurposing.

## 2. Repurposing of Human Kinase Inhibitors as Antimicrobials

### 2.1. Human Kinase Inhibitors Designed as Eukaryotic Competitive Ligands for Protein Kinase ATP Binding Sites 

Since the advent of complete bacterial genomic sequencing in the mid-1990s, attempts at finding novel antibacterial agents in the pharmaceutical industry have been generally directed toward three discovery approaches: new molecular targets, found by comparative genomics; new structures for old molecular targets; and cell-based screening for novel structures. Unfortunately, efforts to screen existing synthetic chemical libraries designed mainly for eukaryotic targets during the era of the great expectations of comparative genomics had been unproductive [11,12]. However, more recently a Pfizer team evaluated the company screening library of 1.6 million compounds as antimicrobials, with the focus on Gram (−) organisms [13]. A high percentage of the compounds in this library were designed to function as eukaryotic protein kinase competitive ligands for the ATP binding site. Initial drug screening utilized an *Escherichia coli* strain that was membrane-compromised and efflux pump-deficient strain, as well as *Hemophilus influenzae*, *Moraxella catarrhalis*, and *Staphylococcus aureus.* Several of the pyridopyrimidines **1**–**3** (Table 1), which were derived from pharmacophore **4** (PD173074; Table 1), a protein kinase inhibitor, exhibited activity against the organisms [13]. They are bactericidal to *E. coli*, and several other pathogens (Table 1) and showed improved activity in combination with other antibacterial agents. Notable synergy with compound **1** (Table 1) were triclosan and ciprofloxacin against *H. influenzae* (Table 1). The Pfizer team also determined the target of the active structures that affects the first committed step in fatty acid (FA) biosynthesis, i.e., the acetyl-CoA carboxylase biotin carboxylase (BC) subunit. This finding establishes the functionality of targeting the fatty acid biosynthetic pathway and as a source of potential antimicrobial targets clarifies the synergistic interaction of compound **1** with triclosan, another fatty acid synthesis targeting drug [13]. This project reignited the hope for exploration of available small molecule libraries which target eukaryotic enzymes for use against pathogens, thereby initiating drug repurposing on a higher scale.

In the decade following the initial efforts by the Pfizer team, several other HumKIs having the 2-aminopyrimidine and/or pyridine moieties demonstrated antibacterial activities. Compound **5** (Table 1), a known p38 HumKI [14,15] was evaluated against a methicillin-resistant *S. aureus* (MRSA) strain carrying the BlaR1 (also known as MecR1) protein [16]. BlaR1 is a β-lactam antibiotic sensor/signal transducer gene repressor protein for the bla operon which is responsible for inducible β-lactam resistance leading to expression of the class A β-lactamase PC1 and/or the penicillin-binding protein 2a (PBP2A) [16]. This contrasts with the primary resistance mechanism typical in MRSA resistance which utilizes intrinsically β-lactam resistant PBP2A. A key point for targeting BlaR1 is that it experiences phosphorylation at a minimum of one serine and one tyrosine in the cytoplasmic domain on exposure to β-lactam antibiotics [16]. This activity suggests that phosphorylation by small molecules could reverse the methicillin-resistant phenotype, resulting in a return to β-lactam antibiotic susceptibility. Based on this hypothesis, a library of 80 known kinases was screened. Of those tested, compound **5** (Table 1) was shown to lower the MICs of oxacillin, without having intrinsic anti-MRSA activity (its MIC alone—≥64 μg/mL). HumKI **5** demonstrated a reproducible decrease by a factor of four in the MIC of oxacillin for *S. aureus* MRSA252 at 7 μg/mL [16]. Structural modification of compound **5** (Table 1) by replacement of the hydroxy group of the phenol with the (un)branched alkyl groups led to compounds **5a**–**5c** (Table 1) which exhibited activity against several other MRSA strains lowering the MICs of oxacillin to 7 μg/mL [16]. This finding that the MRSA phenotype can be reversed by small molecules offers a new strategy for expanded reuse of the β-lactam antibiotics.

Another representative of HumKIs evaluated against MRSA strains is pyrazolopyridazine compound **6** (GW779439X; Table 1). Compound **6** is a known HumKI, designed as a CDK4 inhibitor. [17]. Unfortunately, as a HumKI, it lacked specificity while maintaining high levels of toxicity. When repurposed as an antibacterial, GW779439X was reported to enhance the response of MSSA and MRSA isolates to a variety of β-lactam antibiotics via PASTA kinase Stk1 (eukaryotic-like serine/threonine protein kinases—eSTKs) inhibition [18]. This adjuvant activity has also been reported for the sulfonamide class of HumKIs [19]. The PASTA kinases are a highly conserved family of bacterial kinases that exhibit homology with eukaryotic kinases. PASTA kinases have central roles in different bacterial pathways including basic metabolism, virulence factor expression, and antibiotic resistance mechanisms, e.g., β-lactam susceptibility. Genetic deletion of PASTA kinases in numerous Gram (+) organisms leads to their re-sensitization to β-lactam antibiotics [20,21]. Several compounds were synthesized using the GW779439X (compound **6,** Table 1)-type scaffold, which exhibited adjuvant activity with ceftriaxone against a wild-type MRSA strain (LAC), but with altered substituents on the phenyl ring. Of those, CAF078, (compound **6a**, Table 1), in which the CF_3_ group of **6** has been replaced by a cyano- group, had a potency similar to compound **6** (Table 1); both compounds have the ability to completely inhibit the bacterial growth at submicromolar concentrations [18].

Another HumKI having the amimopyrimidine moiety that has been evaluated for its antimicrobial activity against *S. aureus* [22] is Ceritinib (compound **7**; Table 1), a tyrosine kinase inhibitor used in cancer treatment to inhibit anaplastic lymphoma kinase (ALK). The biochemical evaluation of Ceritinib against both Gram (−) and Gram (+) bacteria was based on a high throughput screen(s). Ceritinib demonstrated antimicrobial activity only against Gram (+) bacteria, with the best activity against *S. aureus* laboratory and clinical MRSA and MSSA isolates (MICs and MBCs of 8–16 μg/mL and 8–64 μg/mL, respectively) [22]. Further examination of Ceritinib’s activity against biofilm formation showed significant biofilm mass reduction (of more than 2 log10 at 32 μg/mL) as well as eradication of the preformed biofilm. In addition, disruption of bacterial membrane was observed, leading to the induction of oxygen radicals in the presence of Ceritinib. It is also bactericidal to *S. aureus* persisters at 1 × MIC in a dose-dependent manner and ameliorates infection in subcutaneous abscesses of the mouse model [22]. Unfortunately, the *S. aureus* tyrosine kinases and eucaryotic kinases are structurally disparate; therefore, the mechanism of action of this HumKI in *S. aureus* has not yet been elucidated [22].

The same team that reported the GW779439X (compound **6,** Table 1) effect on *S. aureus* has been studying procaryotic kinases, including PASTA kinases [23], and evaluating kinase inhibitors. These studies included probing the effect of HumKI GSK690693 (compound **8**, Table 1), an inhibitor of AKT enzyme [17,24], on the sensitization of bacteria to β-lactam antibiotics [25]. Although compound **6** (GW779439X; Table 1) had very low activity against *S. aureus* strains [18,25], it exhibited selective activity against *Listeria monocytogenes’* PASTA kinase PrkA [25]. In addition, GSK690693 is relatively selective for isoforms of AKT eukaryotic kinases (38, 40, 57) in addition to highly similar *S. aureus* and *L. monocytogenes* kinases [25]. In continuation of their work, the research team expanded their evaluation of the imidazopyridine aminofurazans (e.g., compound **8**, and **8a**-**8c**, Table 1) to other bacterial species [26]. The choice for the latter was based on those microorganisms having kinases that have a high degree of similarity with the eukaryotic-like Ser/Thr kinases (eSTKs). One such enzyme is the actinomycetes’ PknB, and eSTK which is essential for mycobacteria. Computational modeling was used to identify HumKIs that bind to PknB, and the selection for further investigation was based on the hits having drug-like characteristics that were expected to have an increased likelihood of cell entry [26]. Based on the latter consideration (characteristics for bacterial cell entry), in addition to compound **8** (GSK690693, Table 1), several other compounds, including **8a**–**8c** (Table 1) were considered for biochemical evaluation [26]. Ki values for all four compounds (**8**, **8a**–**8c**; Table 1), albeit similar were significantly better than the remainder of the compounds tested. These compounds substantially inhibit the growth of nonpathogenic mycobacteria (Msmeg), four pathogenic mycobacterial strains (Mtb, Mche, Mab, and *Mycobacterium marinum* (Mmar)), and the pathogenic nonmycobacterial actinobacteria *Nocardia asteroids* (Nast) alone, as well as at micromolar concentrations when used together with a β-lactam for most of the aforementioned mycobacterial species [26].

While their previous work primarily focused on the effectiveness of compound **6** (GW779439X Table 1) against *S. aureus* [18], an in-silico evaluation of this compound against the PknB of Mtb has also been performed [18]. Subsequently, the team identified compound **6** (Table 1) as a biochemical inhibitor of Mtb PknB23 with a Ki of 420 nM [27]. To improve the bacterial target specificity of new PknB inhibitors, efforts have been focused on finding PknB inhibitors with less human kinase activity. More than 100 compounds were evaluated in silico, from which 17 compounds were chosen based on their predicted reduced specificity toward the human kinase and synthetic feasibility [27]. Of these, 4 compounds, with a differently substituted phenyl substituent, such as in compound **9** (Table 1), were microbiologically active (≤19 μM) and more PknB-specific, relative to the human kinase. Even though compound **9** did not have the lowest MIC values of the 4 compounds and has a comparable cytotoxicity to compound **6** (due to the presence of NO_2_ group), it had the best reductions in off-target effects, while maintaining PknB/Mtb activity [27]. These results are encouraging for the possibility of the development of a combination therapy against Mtb, especially in the light of the recently published results from a screen of 8900 β-lactams by a consortium of academic and governmental, as well as private commercial companies, which identified nearly 1600 lactams with anti-Mtb activity [28].

**Table 1:** Examples of human kinase inhibitors designed as ATP-ligands with antimicrobial activity and potentiation of the activity of β-lactam antibiotics.

Bacteria	Eukaryotic Kinase/Inhibitor/Type of Inhibition/Ref.	Bacterial Target of HumKI/ MOA/Ref.	Type of Antibacterial Activity/Adjuvant/Ref.
*M. catarrhalis*, *S. aureus*, *E. coli*, *H. influenzae*	Compound **4** (**PD173074**) is an inhibitor of VEGFR2 and FGFR1 kinases. 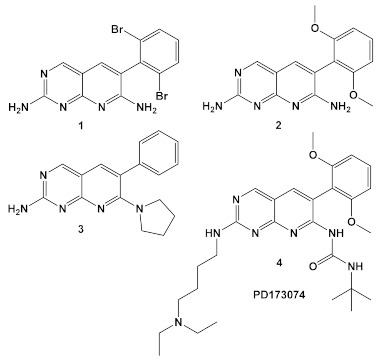 [13]	biotin carboxylase ATP-binding. [13]	Bactericidal alone in vitro & in vivo. Adjuvant activity of compound **1** with triclosan and ciprofloxacin in *H. influenzae* [fractional inhibitory conc. (FIC) 0.37 and 0.26, respectively)] [13]
*S. aureus* MRSA	Inhibitor of the p38 serine/threonine kinase signal-transducing enzyme. 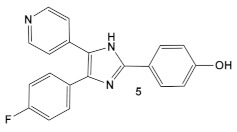 [14,15] 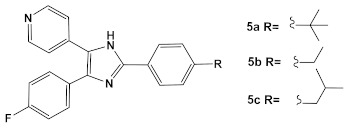 [16]	Phosphory- lation of BlaR1. [16]	Reversing of the MRSA phenotype which makes is susceptible to β-lactam antibiotics [16] **5a**–**5c** potentiate oxacillin at 7 μg/mL against MRSA strains
*S. aureus* Multiple MRSA and MSSA isolates	**GW779439X** putative human CDK4 inhibitor with low specificity and high toxicity. 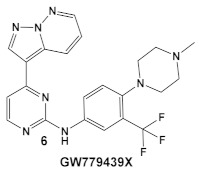 [17] Scaffold modifications of HumKI **GW779439X** (**6**) leads to an STK1 inhibitor **CAF078** (**7**) which retains the **GW779439X** activity. 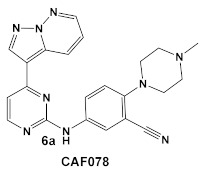 [18]	Inhibition of StK1 in *S. aureus* both MSSA and MRSA strains [18]	Potentiation of β-lactam antibiotics against MRSA by increasing β-lactam susceptibility 2−512-fold—mainly nafcillin and oxacillin, to a lesser degree ceftriaxone [18]
*S. aureus*	**Ceritinib** ATP-competitive tyrosine kinase (ALK) inhibitor 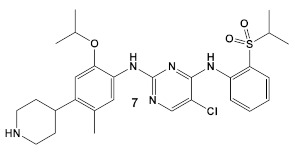 [19]	MOA unknown Disrupts bacterial cell membrane. [20]	Anti-MRSA persisters & antibiofilm mass at a range of 8–16 μg/mL [22]
*L. monocy-* *togenes*	**GSK690693** is an AKT inhibitor. 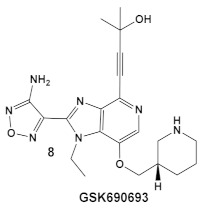 [17,25,26]	Targets *L. monocytogenes* PASTA kinase PrkA [25]	Antimicrobial activity alone, as well as potentiation of β-lactam antibiotics. [25]
*Mycobacteria: M. smegmatis, M. bovis, Mtb and Nocardia asteroides*	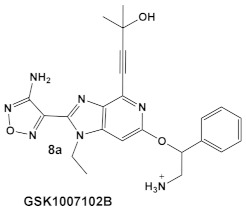 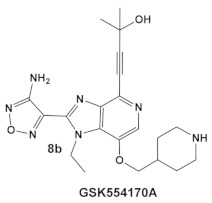 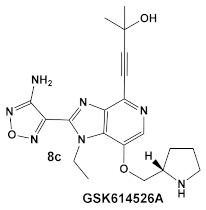 [17,26]	GSK690693 binding to PknB in an ATP-competitive fashion. [26]	Potentiation of β-lactam antibiotics—mainly nafcillin and oxacillin, to a lesser degree ceftriaxone [26]
*M. tuberculosis*	**GW779439X** putative human CDK4 inhibitor with low specificity and high toxicity. 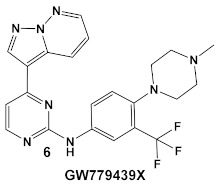 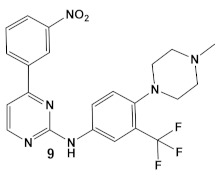 [17,18,27]		

### 2.2. Human Kinase Inhibitors Designed as Ligands of the Catalytic and Non-Catalytic Cys Residues of Eukaryotic Protein Kinases

Modification of both catalytic and non-catalytic cysteine (Cys) residues have been extensively explored in drug discovery. Targeting the Cys in the enzyme active site (the catalytic cysteines) will undoubtfully have an impact on enzyme function, e.g., the inhibitors of the catalytic Cys of deubiquitinases and caspases. Unfortunately, selectivity of an inhibitor, in most cases, is difficult to achieve, since these catalytic cysteines residues are highly conserved within the enzyme families/isoforms. The advantage/attractiveness of targeting the non-catalytic cysteines is that they are generally less conserved, which could allow for selective target binding. Chemical proteomics utilizing activity-based probes have identified a variety of non-catalytic Cys, whose function has been modulated in human kinases. With the development of Cys-active compounds, the view for covalently bound drugs has also evolved as reflected in the number of HumKI targeting non-cat Cys approved by FDA [29].

Afatinib, compound **10** (Table 2), which inhibits the ErbB family of human tyrosine kinases [29], is one of the first covalent irreversible binders to be examined for its activity against bacterial biofilm, which is a subject of great interest and intense research [30,31,32]. In the search for an antibiofilm agent, afatinib was chosen to be evaluated against Gram (+) and Gram (−) organisms, however, it showed no activity beyond that of its solvent DMSO. Therefore, the authors contributed the antibiofilm reduction, to the DMSO [30].

Dacomitinib, compound **11** (Table 2), an epidermal growth factor receptor tyrosine kinase (EGFR), exhibits antibiofilm activity [33]. The bacterial target of this study is the FtsZ protein (filamentous temperature-sensitive mutant Z), the first protein recruited to the division site in bacterial cell division. It is considered to be a potential target for new antimicrobials, due to (1) its ability to inhibit bacterial cell division; (2) the fact that it is conserved in a high percentage of bacterial species; and, (3) the fact that it is absent in eukaryotes, although FtsZ does share up to 50% of the sequence identity with tubulin [33]. Further work done by these researchers, using virtual screening, identified new inhibitors of FtsZ against representatives of both Gram (+) and Gram (−) bacterial species, *B. subtilis* and *S. aureus* as well as *E. coli*, respectively, from compound libraries. Dacomitinib (compound **11**, Table 2) was found to be a potential inhibitor of FtsZ. The proposed binding mode for Dacomitinib in FtsZ places it in the PC (PC 190723) [34], a known inhibitor of the FtsZ,22 binding pocket, where Asp199 and Thr265 are expected to interact with it through the hydrogen bond- and *π*-anion interactions.

This finding was further confirmed by in vitro and in vivo bioassays, which showed the promise of Dacomitinib against *B. subtilis* and MSSA and MRSA strains, with the best antimicrobial activity against MRSA strains [33]. Dacomitinib also has activity against *E. coli*, but only in the presence of compounds that have the ability to increase the permeability of the outer membrane [33]. Therefore, modifications in compound **11** (Table 2) directed towards increasing its water solubility are expected to improve its antimicrobial activity [33].

Bay 11-7085 (compound **12**, Table 2) and *Bay 11-7082* (compound **13**, Table 2) inhibit IκB-α dissociation from NF-κB, thereby blocking TNFα-induced phosphorylation of IκB-α and reducing inflammation [35]. At 10 µM (2.5 μg/mL), compound **12** irreversibly inhibits IκB-α phosphorylation resulting in anti-inflammatory efficacy in both the rat adjuvant arthritis and Carrageenan rat paw edema model systems [35]. In addition to its anti-inflammatory effects, compound **12** also induces cancer cell apoptosis through the inhibition of NF-κB signaling. In addition, it has NF-κB -independent anti-cancer activity [36]. **Bay 11-7082** (compound **13**; Table 2), has the same scaffold as Bay 11-7085 but instead of the tert-butyl group in the phenyl ring, Bay-11-7082 has a methyl group. Compound **13** (Bay 11-7082; Table 2) has been proposed to have, in addition to the aforementioned activities of Bay 11-7085 (compound **12**), inhibitor activity against mammalian protein tyrosine phosphatases (PTPs), which regulate various cellular processes [37]. This finding prompted the evaluation of compound **12** (Table 2) for potential activity against bacteria and fungi, e.g., *Candida* species, even though the bacteria lack NF-κB signaling [38]. Bay 11-7085 demonstrated in vitro and in vivo bactericidal activity against MDR *S. aureus* with a MIC of 4 μg/mL [38]. The authors also performed biofilm studies, which demonstrated that Bay 11-7085 is inhibitory to *S. aureus*—*Candida* spp. polymicrobial biofilm formation [38].

Due to its anti-staphylococcal activity, Bay 11-7082 (compound **13**, Table 2) medicinal chemistry protocols were used towards structure optimization [39]. From the library of compounds synthesized by the authors, which included replacement of the cyano moiety, varying the substituents on the phenyl ring, and removing the double bond next to the sulfone, thereby eliminating its Michael acceptor activity, only the replacement of the tert-butyl group with the F-atom on the phenyl ring retained the inhibitory activity of Bay 11-7082 towards *S. aureus*. Further synthetic efforts were directed toward replacing the phenyl ring with the pyrazine ring (compounds **14**–**16**, Table 2) while retaining the cyano-group, but not as part of the α, β-unsaturated system, to remove the compounds’ ability to act as Michael acceptors. The latter was an attempt to reduce the possible off-target binding (toxicity) of the original compound **13** (Bay 11-7082) [39]. The preparation of these latter compounds (**14**–**16**, Table 2) was inspired also by the earlier report for sulfone **14** (Table 2) having anti-staphylococcal activity [40]. Of these, compounds **15** and **16** are the most promising compounds as adjuvants for Penicillin G (Pen G), since they can potentiate Pen G activity against MRSA, shifting the MIC 3.74 to 0.39 μM, a value that is comparable to the reduction of the MIC by Bay 11-7082 (MIC of penicillin G from 3.74 to 0.23 μM, a 16-fold reduction). The authors assumed that both compounds (**15** and **16**; Table 2) would not act as Michael acceptors [39].

**Table 2:** Representative examples of Human Kinase Inhibitors designed as irreversible covalent ligands (Michael acceptors) of the non-catalytic Cys in a eukaryotic protein kinase with antimicrobial activity and potentiation of the activity of β-lactam antibiotics.

Bacteria	Eukaryotic Kinase/Inhibitor/Type of Inhibition/Ref.	Bacterial Target of HumKI/ MOA/Ref.	Type of Antibacterial Activity/Adjuvant/Ref.
*E. coli*, *P. aeruginosa* & *Salmonella typhimurium*	**Afatinib**/FDA appr. 2013 EGF receptor and ErbB tyrosine kinase inhibitor 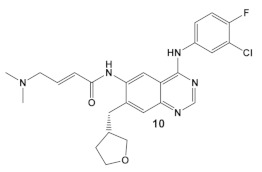 [9,29]	Specific target/MOA unknown. [30]	Bactericidal to biofilm cells.
*B. subtilis*, *S. aureus*, MSSA, and MRSA strains.	**Dacomitinib**/FDA appr. 2018 EGFR tyrosine kinase inhibitor 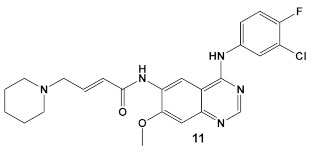 [29]	FtsZ protein (filamentous temperature-sensitive mutant Z) via hydrophobic and H-bonding interactions. [33]	Inhibition of bacterial cell division. MICs 16 μg/mL against *B. subtilis* 168 and 32–64 μg/mL against *S. aureus* strains. [33]
*S. aureus*, MSSA, *Candida albicans*	**BAY 11-7085**, TNF-α-stimulated IκBα phosphorylation inhibitor 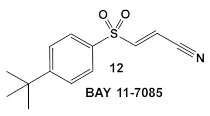 [35,36]	Specific target/MOA unknown. [38]	Antibiofilm as monoculture and polymicrobial co-culture. *S. aureus* (MRSA, 4 μg/mL) and *Candida albicans*/anti-biofilm. [38]
*S. aureus* MRSA	**BAY 11-7082**, IκBα phosphorylation and NF-κB inhibitor. IκB kinase and protein tyrosine phosphatases (PTPs) active site inhibitor 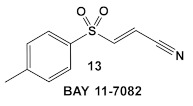 [35,36] 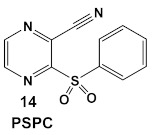 [40] 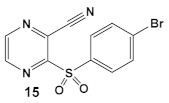 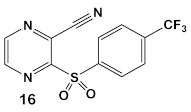 [39]	MOA undetermined [39]	Bactericidal alone and in combination Compound **13** adjunctive for penicillin G. (16-fold reduction in MIC). For compounds **15** and **16** the reduction of penicillin G MIC to 0.39 μM. Modest antimicrobial activity against Gram (−), e.g., *P. aeruginosa* [39]

### 2.3. Human Kinase Inhibitors of Amide and Urea Chemotypes

The multikinase inhibitor, *Sorafenib* (FDA approved 2005), compound **17** (Table 3), and its derivative PK-150, compound **18** (Table 3), are anticancer agents, e.g., kinase inhibitors, inhibitors of stearoyl-CoA desaturase 1, as well as inhibitors of the insulin-like growth factor I receptor [41]. Most recently, amide- and urea-based HumKIs have been characterized as antimicrobials. PK150 exhibits both anti-MSSA and anti-MRSA activity. In addition, PK150’s ability to eliminate preexisting biofilms was similar to that of Sorafenib or Regorafenib (3 µM for either Sorafenib/Regorafenib) [42]. In addition, it can inhibit the staphylococcal menaquinone metabolism vis á vi its inhibition of demethylmenaquinone methyltranferase (MenG) biosynthesis. Furthermore, since it induces over-activation of SpsB, a *S. aureus* signal peptidase I enzyme, it is not affected by environmentally-acquired resistance mechanisms [42].

*IMD0354*, amide **19** (Table 3), is an NF-κB inhibitor [43,44,45]. It is another compound with activity against resistant strains of *S. aureus*. It has been identified amongst ~82,000 small molecules as an anti-infective agent that prevents MRSA infection in Caenorhabditis elegans [46]. The protection mechanism by which IMD0354 functions was determined to be a direct antimicrobial activity against MRSA MW2 with a MIC of 0.06 µg/mL. In addition, IMD0354 exhibits bacteriostatic activity against VISA, VRSA, and VRE strains at low concentrations (≤2 µg/mL). Compound **19** (Table 3) has bactericidal activity at high concentrations (≥8 µg/mL) against VISA in a strain-dependent manner. In addition, this compound does not cause any hemolysis in *C. elegans* at concentrations up to 16 µg/mL, or toxicity to *C. elegans* up to 2 µg/mL with 90% *C. elegans* survival at >64 µg/mL [46]. The antimicrobial mechanism of IMD0354 when applied in high concentrations ≥ 4 µg/mL was identified by the authors as membrane permeabilization [46]. It is interesting to note that the antimicrobial activity of IMD034 surpasses its anti-cancer activity [46].

IMD0354, compound **19,** and OSU-03012, compound **20** (Table 3), a PDK1 inhibitor [47], potentiate the antimicrobial activity of colistin in Gram (−) bacteria [48]. Four Gram (−) isolates, i.e., *E. coli*, *K. pneumoniae*, *A. baumannii*, and *P. aeruginosa*, were evaluated for the screening of a 942-compound library of known Kis. From the ~50 hits, the compounds chosen for further evaluation were compound **19** and compound **20** (Table 3). Compound **19** (IMD-0354; Table 3) demonstrated potent and consistent activity against the colistin resistant strains, for both colistin in the chromosomally encoded colistin resistant strains, as well as strains containing the *mcr-1* plasmid-borne colistin resistance gene [48]. Its strongest activity was against *A. baumannii* and *K. pneumoniae*, with limited activity against *P. aeruginosa*. Compound **20** (Table 3) also had significant activity against the aforementioned isolates. The MOA of compound **19** (Table 3) is associated with the suppression of lipid A modification in colistin resistant strains. Further testing showed that compound **20** (OSU-03012, Table 3) exhibited ubiquitous activity in a diverse array of susceptible Gram (−) bacilli [48]. Compounds **19** and **20** alone at their respective active concentrations, do not inhibit bacterial growth, while both clearly elicit cell death upon co-treatment with colistin [48].

## 3. Summary

Kinases with about 518 members are the second largest family of drug targets [49]. HumKIs are now a well-established class of anti-cancer agents [7,8,9]. Development of drug resistance during kinase-inhibitor therapy is also common. As mentioned earlier the pyridopyrimidines are representative examples of the first small heterocyclic molecules designed and some were later developed as ATP-competitive ligands for the ATP binding site of HumKI. For the past two decades, after the FDA approval of imatinib in 2001, HumKIs have been successfully used clinically. However, challenges in kinase drug discovery exist, such as acquired drug resistance, which mainly stems from the targeting of the conserved ATP-binding site of kinases (Table 1) [7,8,9].

To get around the high level of drug resistance associated with ATP-binding sites, covalent inhibition of the kinase non-catalytic Cys residues was targeted. This target has the advantage of being less conserved, which allows for selective target binding, and subsequent lowering of the toxicity [50]. Some of the best electrophiles for this type of inhibition of the α, β-unsaturated systems (Michael acceptors) which can bind a known and biologically uncommon target, reducing the likelihood of irreversible covalent off-target binding [27,29]. However, this selectivity has proved difficult to achieve; thus, the current interest is directed toward developing of reversible covalent inhibitors [29]. The move from irreversible to reversible Michael acceptors has been achieved by introducing an electron withdrawing group (e.g., cyano-group) as part of the α, β-unsaturated system [51]. That shift in the electrophilic moiety could be seen in the most recent kinase inhibitors as compared to their earlier counterparts (Table 2) [29]. Mutations at the targeted cysteine site, which exacerbates clinical resistance to even the newer HumKis, might be difficult to overcome since due to the unknown effect the mutation may have on ATP-binding affinity [29].

There are numerous examples across the different chemotypes of the HumKIs in the different repurposing evaluations of kinases as antimicrobials that potentiate the antimicrobial activity of clinically relevant antibiotics against both Gram (+), in most cases against *S. aureus*, and to a lesser extend against Gram (−) bacteria. In most cases, β-lactams have been the chosen antibiotics for rejuvenation, which is to be expected given the fact that the β-lactams enjoy very good pharmacodynamics/pharmacokinetics and low toxicity to humans.

Taken together, it appears that the boundaries of the traditional classification of antimicrobial and anticancer agents are beginning to blur. Repurposing of the existing drugs as antimicrobials is a great opportunity, as well as a great challenge. The early examples of the HumKIs were developed through rational drug design. Recently, the high throughput evaluation of future lead molecules has been the most popular approach, as demonstrated in most of the examples of drug repurposing described here. This will be the expected type of drug discovery going forward, since thousands of structures of kinase modulators are publicly available and accessible through different compound libraries. An increasing understanding of kinase biochemistry and the new drug design technologies (Cheminformatics, AI) will hopefully provide the platform to enable us to grasp the complexity of enzyme/receptor modulations [52,53,54].

## Figures and Tables

**Table 3:** Representative examples of amide- and urea-based human kinase inhibitors of eukaryotic protein kinase with antimicrobial activity and potentiation of the activity of colistin.

Bacteria	Eukaryotic Kinase/Inhibitor/Type of Inhibition/Ref.	Bacterial Target of HumKI /MOA/Ref.	Type of Antibacterial Activity/Adjuvant/Ref.
*S. aureus* MRSA	**Sorafenib**, inhibits multiple targets including Raf serine/threonine kinases, vascular endothelial growth factor receptor tyrosine kinases; VEGFR-1, VEGFR-2, VEGFR-3 and platelet-derived growth factor receptor β (PDGFR-β). 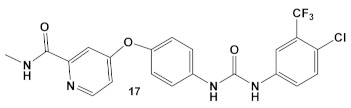 [41] 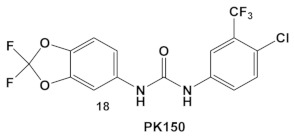 [42]	Inhibitors of MenG biosynthesis Sorafenib exhibits anti-MRSA, including persisters and biofilm-embedded cells, activity. [42]	Anti-persisters and antibiofilm. **PK150** exhibits activity against several pathogenic isolates at submicromolar conc. [42]
*S. aureus* MRSA and VRSA strains	An IKK-β inhibitor 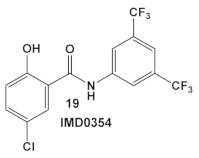 [43,44,45]	MOA at low conc. ≥ 4 µg/mL is membrane permeabilization. MOA at lower conc. is unidentified. [46]	Anti-VRSA activity (MIC 0.06 µg/mL) and inhibition of VRSA adherence and subsequent biofilm formation at sub-MIC levels [46]
*E. coli*, *K. pneumoniae*, *A. baumannii* and *P. aeruginosa*	An IKK-β inhibitor 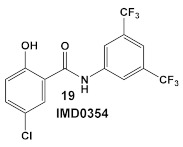 [43,44,45] A pyruvate dehydrogenase kinase-1 (PDK-1) 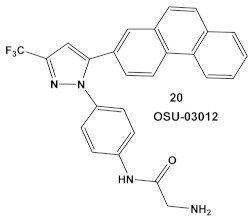 [47]	Suppression of lipid A modification in colistin-resistant strains, an insight into its MOA. [48]	Potentiation of colistin against Gram (−) bacteria. [48]

## Data Availability

Not applicable.

## Abbreviations

AKT	alpha serine/threonine-protein kinase
ALK	anaplastic lymphoma kinase
ATP	Adenosine triphosphate
BLAR1	cell-surface receptor protein (*S. aureus*)
CDK4	cyclin-dependent kinase 4
EGF	epidermal growth factor
EGFR	epidermal growth factor receptor
ErbB	receptor tyrosine kinase
FA	Fatty acid(s)
FGFR1	fibroblast growth factor receptor 1
FtsZ	filamenting temperature-sensitive mutant Z
HumKIs	Human Kinase Inhibitors
IκB-α	nuclear factor of kappa light polypeptide gene enhancer in B-cells inhibitor, alpha
IKK-β	inhibitor of nuclear factor kappa-B kinase
MBC	minimum bactericidal concentration
MDR	multi drug resistant
MEK1/2	Mitogen-Activated protein kinases (also known as MAPKK and MAP2K0
MenG	demethylmenaquinone methyltransferase
MIC	minimum inhibitory concentration
MOA	mechanism of action
Mtb	*M. tuberculosis*
NF-κB	nuclear factor kappa B protein transcription factor
NP	Nanoparticles
PASTA	serine/threonine kinase-associated kinases
PBP2A	penicillin binding protein 2a
PknA and PknB	protein kinases A and B (*M. tuberculosis*)
PrkA	putative serine protein kinase
PKI	protein kinase inhibitors
ROS	reactive oxygen species
eSTKs	eukaryotic-like Ser/Thr kinases
Stk1	serum thymidine kinase 1
TNF-α	tumor necrosis factor, alpha
TK	Tyrosine kinase
VEGFR2	Vascular endothelial growth factor receptor 2
Bacterial Names	
*A. baumannii*	*Acinetobacter baumannii*
*B. subtilis*	*Bacillus subtilis*
*E. coli*	*Escherichia coli*
*H. influenzae*	*Haemophilis influenzae*
*K. pneumoniae*	*Klebsiella pneumoniae*
*M. catarrhalis*	*Moraxella catarrhalis*
Mtb, *M. tuberculosis*	*Mycobacterium tuberculosis*
*M. bovis*	*Mycobacterium bovis*
*M. smegmatis*	*Mycobacterium smegmatis*
MRSA	methicillin-resistant *Staphylococcus aureus*
MSSA	methicillin-susceptible *S. aureus*
*N. asteroides*	*Nocardia asteroides*
*P. aeruginosa*	*Pseudomonas aeruginosa*
*S. aureus*	*Staphylococcus aureus*
VISA	vancomycin-intermediate *S. aureus*
VRSA	vancomycin-resistant *S. aureus*
VSSA	vancomycin-susceptible *S. aureus*
VRE	vancomycin-resistant *Enterococcus*
